# Trends in the risk of second primary malignancies among survivors of chronic lymphocytic leukemia

**DOI:** 10.1038/s41408-019-0237-1

**Published:** 2019-09-30

**Authors:** Vivek Kumar, Sikander Ailawadhi, Leyla Bojanini, Aditya Mehta, Suman Biswas, Taimur Sher, Vivek Roy, Prakash Vishnu, Julian Marin-Acevedo, Victoria R. Alegria, Aneel Paulus, Sonikpreet Aulakh, Madiha Iqbal, Rami Manochakian, Winston Tan, Asher Chanan-Khan, Meghna Ailawadhi

**Affiliations:** 1 Department of Medicine, Harvard Medical School, Boston, MA USA; 2 Division of Hematology-Oncology, Mayo Clinic, Jacksonville, FL USA; 3 Department of Medicine, Mayo Clinic, Jacksonville, FL USA; 4 Department of Cancer Biology, Mayo Clinic, Jacksonville, FL USA

**Keywords:** Chronic lymphocytic leukaemia, Risk factors

## Abstract

With improving survivorship in chronic lymphocytic leukemia (CLL), the risk of second primary malignancies (SPMs) has not been systematically addressed. Differences in risk for SPMs among CLL survivors from the Surveillance, Epidemiology, and End Results (SEER) database (1973–2015) were compared to risk of individual malignancies expected in the general population. In ~270,000 person-year follow-up, 6487 new SPMs were diagnosed with a standardized incidence ratio (SIR) of 1.2 (95% CI:1.17–1.23). The higher risk was for both solid (SIR 1.15; 95% CI:1.12–1.18) and hematological malignancies (SIR 1.61; 95% CI:1.5–1.73). The highest risk for SPMs was noted between 2 and 5 months after CLL diagnosis (SIR 1.57; 95% CI:1.41–1.74) and for CLL patients between 50- and 79-years-old. There was a significant increase in SPMs in years 2003–2015 (SIR 1.36; 95% CI:1.3–1.42) as compared to 1973–1982 (SIR 1.19; 95% CI:1.12–1.26). The risk of SPMs was higher in CLL patients who had received prior chemotherapy (SIR 1.38 95% CI:1.31–1.44) as compared to those untreated/treatment status unknown (SIR 1.16, 95% CI:1.13–1.19, *p* < 0.001). In a multivariate analysis, the hazard of developing SPMs was higher among men, post-chemotherapy, recent years of diagnosis, advanced age, and non-Whites. Active survivorship plans and long-term surveillance for SPMs is crucial for improved outcomes of patients with a history of CLL.

## Introduction

Chronic lymphocytic leukemia (CLL) is the most common leukemia diagnosis in adults^1^, with favorable outcomes in majority of patients and a 5-year survival of ~85%^2^. A large proportion of CLL patients may never require treatment, requiring only ongoing surveillance. Furthermore, in recent years there have been several advancements in the management of CLL with the result that even those patients who do require treatment over their lifetime, are now living longer with better disease control^2,3^. As the population of CLL survivors grows, there is a need to understand their long-term health. It is known that nearly one in five cancers diagnosed currently occurs in an individual with a previous diagnosis of cancer, and these second primary malignancies (SPMs) are a leading cause of morbidity and mortality among cancer survivors^4^. Thus, understanding the risks and trends in incidence of SPMs in cancer survivors, especially for primary malignancies like CLL where patient outcomes are relatively good, is very important in order to balance survivorship with the morbidity and mortality from SPMs.

Prior studies have looked at the risk of SPMs in patients with CLL but have not reported trends over time or compared the risk of SPMs among CLL patients with that of the general population^5–7^. Most of these studies had small cohorts of patients with short follow-up. The etiologies for SPMs among CLL patients appear to be multifactorial, including a dysregulated immune system, shared environmental and genetic risk factors, and detection bias due to increased surveillance in patients with a primary cancer diagosis^8^. The resulting immunodeficiency due to CLL and/or its treatment may play a vital role in developing SPMs among these patients as the spectrum of SPMs is similar to patients who have undergone renal transplant^9,10^. Indeed, chemoimmunotherapy used in CLL treatment e.g., a combination of fludarabine, cyclophosphamide, and rituximab (FCR) has been associated with a 2.38 times higher risk of developing SPMs^11^.

Using the Surveillance Epidemiology and End Results (SEER) database, we conducted a large, population-based analysis of SPMs occurring in CLL patients to better understand their trends and document the risk of specific SPMs according to patients’ demographic factors. This will help with a better understanding of the morbidity associated with SPMs among patients with CLL, and plan optimal utilization of healthcare resources.

## Methods

### The SEER database

We extracted data from the National Cancer Institute’s SEER database, an authoritative source of population-based cancer statistics in the United States (U.S.), which collects and publishes information on cancer incidence, patient demographic profile, including race/ethnicity, primary tumor site, SPMs, limited data on clinical and treatment profiles, and survival data on cancer patients in the U.S. The SEER database covers ~34% of the U.S. population^2^.

### Study population

Patients were identified using SEER*Stat, version 8.3.5 multiple primary-standardized incidence ratio (SIR) tool. Eligible patients were ≥15-years-old who were diagnosed with CLL according to SEER’s International Classification of Disease (ICD-O-3) during 1973 and 2015. SEER database categorizes subject age as 5-year groups so patients ≥15-years-old were included to account for those >18 years age. Since CLL has a much higher median age at diagnosis, these categories did not make much of a difference to the final cohort but including ≥15-years-old truly represented adult (>18-years-old) patient population. Data on patient’s demographic profile, date of diagnosis of both the CLL and SPMs, chemotherapy treatment (as yes or no/unknown), types of SPMs, survival status and cause of death were abstracted. Patients were excluded if SPMs were detected within the first 2 months of the diagnosis of CLL to avoid ascertainment bias, or if CLL was not their primary (initial diagnosed) malignancy. The year of diagnosis of CLL was used as surrogate for the evolution of treatment modalities due to the absence of actual treatment data in the SEER database, as has been previously published^12,13^. Owing to lack of specific cutoffs for the calendar years to account for these changes, we studied patients diagnosed with CLL in different decades: 1973–1982, 1983–1992, 1993–2002, and 2003–2015.

### Statistical analysis

The risk of SPMs was evaluated by cumulative person-years (PYs) according to age, gender, and calendar-year from 2 months after the date of CLL diagnosis to the date of diagnosis of SPMs, date of death, date of last follow-up or end of study (31 December, 2015), whichever occurred first. The file on expected SPMs in the general population is incorporated in SEER*stat, version 8.3.5. It is based upon the year 2000 U.S. standard population and has been calculated by multiplying the incidence rates specific for gender, race, attained age, and calendar-year by the specific person-years at risk. We estimated the risk of SPMs relative to the U.S. general population as the standardized incidence ratio (SIR), which is the ratio of observed to expected number of events. The absolute excess rate (AER) per 10,000 PYs was estimated by subtracting the expected events from the observed SPMs and dividing the difference by the number of PYs at risk.

A Poisson distribution of observed SPMs was assumed for the calculation of 95% confidence intervals (CIs). We compared SIRs in different time periods and used the criteria of non-overlapping CI to show statistically significant difference. We also estimated incidence rate ratio (IRR) with 95% CI and two-tailed *p*-values to analyze statistically significant difference over various time periods. A multivariate analysis was applied after fitting development of SPMs in time-to-event model to analyze the effect of year of diagnosis on the development of SPMs independent of age, gender, race/ethnicity, and whether chemotherapy treatment was received.

## Results

**Fig. 1:**
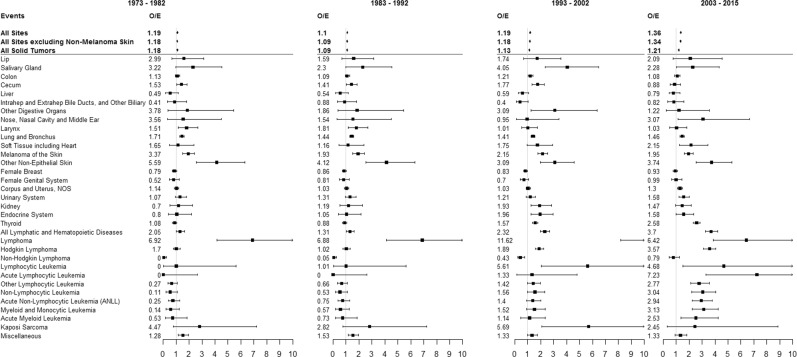
Second primary malignancy (SPM) diagnoses by site, with significantly increased (>1) or decreased (<1) incidence among patients with chronic lymphocytic leukemia (CLL) as the primary malignancy over time in the SEER database

Overall, 38,754 patients with CLL were diagnosed during 1973–2015 and were followed for a total of 269,729 PYs. Select baseline and treatment characteristics for all patients, as well as by year of diagnosis are shown in Table 1. A total of 6487 SPMs were diagnosed among this cohort of CLL patients with a 20% higher risk of developing any SPMs as compared to the U.S. general population (SIR 1.20; 95% CI: 1.17–1.23), attributing 38.85 excess cancers per 10,000 PYs. The increased risk was observed both for solid tumors (SIR 1.15; 95% CI: 1.12–1.87) and hematological malignancies (SIR 1.61; 95% CI: 1.5–1.73). Solid-organ cancers contributed more (67%), while hematological malignancies contributed lesser to the excess risk of SPMs among CLL patients. Among solid-organ cancers, lung/bronchus carcinoma, and melanoma attributed ~59% and 31%, respectively, to the excess risk of SPMs. Looking at individual sites, the highest risk was observed for Kaposi sarcoma (KS) (SIR 3.82; 95% CI: 2.19–6.21), salivary gland tumors (SIR 2.97; 95% CI: 2.13–4.03), skin melanoma (2.14; 95% CI 1.94–2.36), SEER category of skin carcinoma excluding basal and squamous cell subtypes (2.32; 95% CI 2.12–2.53), nose/nasal cavity/middle ear carcinoma (2.16; 95% CI 1.23–3.5), and lip carcinoma (SIR 2.11; 95% CI 1.47–2.93). In contrast the risk of cancers of the tonsils, liver, gallbladder, biliary ducts, female breasts, and corpus uteri was lower among CLL patients than the U.S. general population (Fig. 1). Among hematological malignancies, most of the excess risk was contributed by non-Hodgkin lymphoma (NHL, 23%) followed by Hodgkin lymphoma (HL, 7%) and acute myeloid leukemia (AML, 3%). Since there could be a diagnostic overlap between CLL and the NHL subtype of small lymphocytic lymphoma (SLL), we performed the analysis for hematologic SPMs without including NHL as well. There was still an overall increased risk of hematologic SPMs among CLL (SIR 1.16; 95% CI 1.03–1.47), although the AER was lesser than with NHL included.

**Table 1:** Selected patient and treatment characteristics, overall and by year of diagnosis range

Characteristics	1973–2015 (*N* = 38,754)	1973–1982 (*N* = 6174)	1983–1992 (*N* = 7803)	1993–2002 (*N* = 9018)	2003–2015 (*N* = 15,759)
Person-years	269,729	49,377	67,037	78,891	74,424
Median age (range), years	69 (15, 104)	69 (15, 104)	69 (26, 102)	70 (20, 101)	69 (15, 104)
Age groups, *N* (%)					
15–49 years	2371 (6.12)	332 (5.38)	439 (5.63)	668 (7.41)	932 (5.91)
50–79 years	28,571 (73.72)	4654 (75.38)	5850 (74.97)	6592 (73.10)	11,475 (72.82)
≥80 years	7812 (20.16)	1188 (19.24)	1514 (19.40)	1758 (19.49)	3352 (21.27)
Gender, *N* (%)					
Male	22,964 (59.26)	3617 (58.58)	4593 (58.86)	5336 (59.17)	9418 (59.76)
Female	15,790 (40.74)	2557 (41.42)	3210 (41.14)	3682 (40.83)	6341 (40.24)
Race, *N* (%)					
White	35,179 (90.78)	5744 (93.04)	7192 (92.17)	8255 (91.54)	13,988 (88.76)
Black	2376 (6.13)	375 (6.07)	488 (6.25)	527 (5.84)	986 (6.26)
Other	808 (2.08)	42 (0.68)	100 (1.28)	191 (2.12)	475 (3.01)
Unknown	391 (1.01)	13 (0.21)	23 (0.29)	45 (0.50)	310 (1.97)
Chemotherapy, *N* (%)					
Yes	9988 (25.77)	2504 (40.56)	2923 (37.46)	2330 (25.84)	2231 (14.16)
No/unknown	28,766 (74.23)	3670 (59.44)	4880 (62.54)	6688 (74.16)	13,528 (85.84)
Radiation therapy, *N* (%)					
Yes	494 (1.27)	195 (3.16)	162 (2.08)	88 (0.98)	49 (0.31)
No/unknown	38,260 (98.73)	5979 (96.84)	7641 (97.92)	8930 (99.02)	15,710 (99.69)

### Risk of SPMs by clinical and demographic factors

**Fig. 2:**
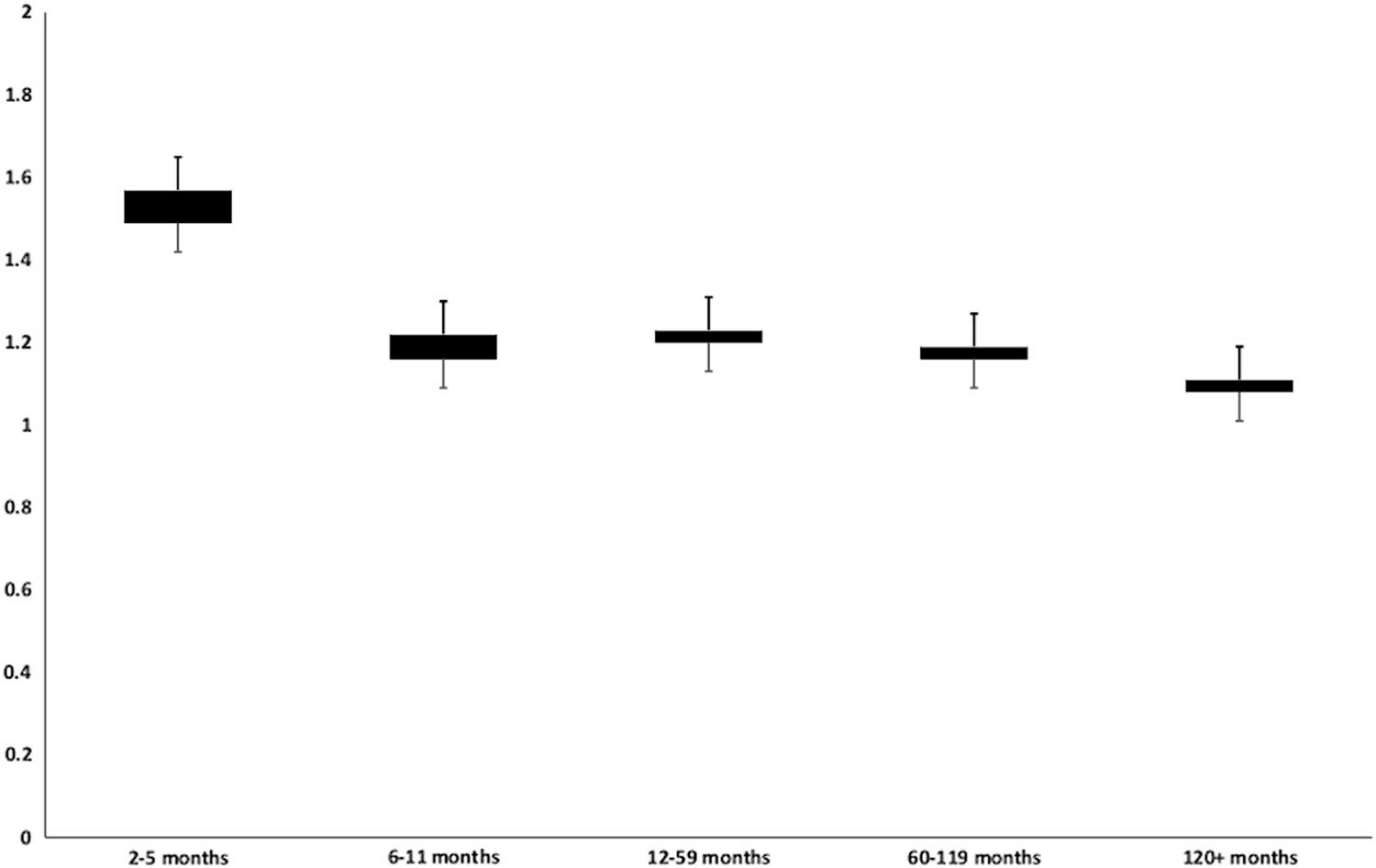
Observed/expected (*O*/*E*) incidence (standardized incidence ratio, SIR) of second primary malignancies (SPMs) by latency period after the diagnosis of chronic lymphocytic leukemia (CLL) over time

**Fig. 3:**
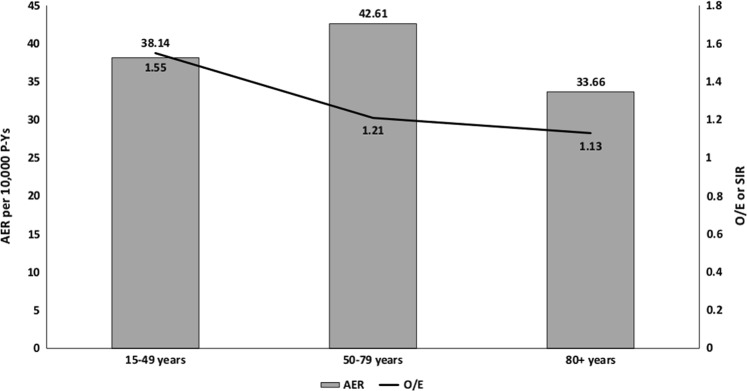
Observed/expected (*O*/*E*) incidence and absolute excess rate (AER) for second primary malignancies (SPMs) by patient age at the time of chronic lymphocytic leukemia (CLL) diagnosis

Exploring the latency of developing SPMs, the maximum risk was seen within 2–5 months after the diagnosis of CLL (SIR 1.57; 95% CI 1.41–1.74). The SIRs decreased significantly after 6 months from the diagnosis of CLL and remained stable during rest of the latency periods (Table 2 and Fig. 2). However, the increase in risk compared to the U.S. population persisted across all latency periods. To further rule out ascertainment bias, we conducted a sensitivity analysis by excluding all SPMs, which were diagnosed within first year after the diagnosis of CLL (Supplementary Table 1). While this excluded many SPMs, the overall SIRs for solid and hematological malignancies did not change significantly. Furthermore, there was no major difference in the trend of SPMs over time. Patients who were followed beyond 10 years after the diagnosis of CLL were at 11% higher risk of developing SPMs as compared to the U.S. population. We noted that the SIRs were highest for CLL patients aged 15–49 years and decreased progressively with patient age (50–79 years and 80 + years). Yet, the highest absolute excess was observed among patients in the age group 50–79-years-old as compared to those between 15 and 49 years or those ≥80-years-old (Fig. 3). For an analysis by race, we noted that the risk of developing any SPM was higher among “other” races (SIR 1.66, 95% CI: 1.37–2) and African Americans (SIR 1.44, 95% CI: 1.29–1.59) as compared to Whites (SIR 1.20, 95% CI: 1.17–1.23). Since the incidence of CLL may be different by race, we analyzed the risk of SPMs in “other” races separately for Asians, Native Americans, and Pacific Islanders as well (Supplementary Table 2). Overall and solid-tumor SPM risk was higher than the general population among all these races. Risk of hematological malignancies was higher only among Asians. Although most of these differences were statistically significant, small sample sizes make further analyses difficult.

**Table 2:** Standardized incidence ratios (SIRs) of second primary malignancies (SPMs) by latency period after the diagnosis of chronic lymphocytic leukemia (CLL), patient age, and race over time

	1973–1982 *O*/*E* (95% CI)	1983–1992 *O*/*E* (95% CI)	1993–2002 *O*/*E* (95% CI)	2003–2015 *O*/*E* (95% CI)
Latency period				
2–5 months	1.61^a^ (1.2–2.11)	1.19 (0.9–1.54)	1.54^a^ (1.23–1.89)	1.79^a^ (1.52–2.08)
6–11 months	1.26 (0.96–1.64)	1.04 (0.8–1.31)	1.02 (0.81–1.26)	1.44^a^ (1.24–1.67)
12–59 months	1.30^a^ (1.18–1.44)	1.17^a^ (1.08–1.28)	1.14^a^ (1.06–1.24)	1.30^a^ (1.22–1.39)
60–119 months	1.11 (0.99–1.25)	1.07 (0.97–1.17)	1.22^a^ (1.13–1.32)	1.34^a^ (1.22–1.46)
≥120 months	1.08 (0.97–1.2)	1.05 (0.96–1.15)	1.18^a^ (1.08–1.29)	1.41^a^ (1.07–1.82)
Patient age				
15–49 years	1.53^a^ (1.17–1.97)	1.31^a^ (1.02–1.67)	1.52^a^ (1.21–1.87)	2.14^a^ (1.6–1.79)
50–79 years	1.18^a^ (1.10–1.26)	1.10^a^ (1.04–1.16)	1.19^a^ (1.13–1.25)	1.37^a^ (1.3–1.44)
≥80 years	1.12 (0.93–1.34)	1.02 (0.87–1.19)	1.04 (0.9–1.2)	1.26^a^ (1.13–1.4)
Patient race				
White	1.19^a^ (1.12–1.26)	1.08^a^ (1.03–1.14)	1.17^a^ (1.12–1.23)	1.36^a^ (1.3–1.43)
Black	1.27 (0.97–1.65)	1.47^a^ (1.18–1.81)	1.49^a^ (1.21–1.8)	1.46^a^ (1.21–1.75)
Others	1.02 (0.33–2.38)	1.62^a^ (1.01–2.45)	1.41 (0.99–1.95)	2.07^a^ (1.55–2.72)

*O*/*E* observed/expected, *CI* confidence interval

^a^Statistically significant

### Trends of SPMs

The risk of developing any SPM remained elevated and was noted to significantly increase during study periods by patient’s year of CLL diagnosis. During 1973–1982 the overall risk was 19% (SIR 1.19, 95% CI: 1.12–1.26) higher as compared to the U.S. population, which increased to 36% (SIR 1.36, 95% CI:1.3–1.42) during 2003–2015 with an IRR of 1.10 (95% CI: 1.02–1.19, *p* = 0.01). This translated to the SPMs attributing to 36 excess cancers per 10,000 PYs during 1973–1982 study period, as compared to 67 excess cancers per 10,000 PYs in 2003–2015 (Fig. 1). The overall elevated risk of SPMs during all study periods was noted both for solid organ as well as hematological malignancies, however, the risk of solid-organ cancers did not change significantly across the study periods, while that for hematological malignancies increased over time, contributing to the overall trend. Solid tumors for which the risk was elevated across all study periods included lung/bronchus carcinoma, salivary gland tumors, melanoma and other skin cancers. Risk for certain other solid-organ cancer types including thyroid and urinary system were significantly higher only in the more recent time period and not previously. Conversely, the risk for solid-organ cancer types, including lip, cecum and KS was higher among CLL patients in the earlier study periods, contributing to the lack of overall significant trend for solid-organ cancers as a whole (Fig. 1).

Looking at them separately, the risk for hematologic SPMs was significantly higher during the study period 2003–2015 (SIR 2.58, 95% CI: 2.32–2.86) as compared to the patients diagnosed during 1973–1982 (SIR 1.08, 1.05–1.34) (Fig. 1). Among these, the risk of HL was higher during all the study periods, while the higher risk of leukemia was observed only in the latter two study periods (1993–2002 and 2003–2015) and not earlier (1973–1982 or 1983–1992) (Fig. 1). In fact, the risk of secondary leukemia was lower than the general population during 1973–1982. During 1993–2002 and 2003–2015 periods, the highest risk of occurrence among leukemia subtypes was observed for AML. The higher risk for NHL as compared to the general population was seen across all study periods.

Owing to unequal follow-up across different study periods, the risk of developing SPMs was stratified according to the latency period after CLL diagnosis. The risk of developing SPMs was consistently elevated during 2–5 months after CLL diagnosis during all the study periods but the risks during other time period were more variable (Table 2 and Fig. 2). However, during 2003–2015, the risk of SPMs as compared to the U.S. population remained elevated for men and women during all the latency time periods, including >10 years after CLL diagnosis. Of note, fewer patients had a follow-up of >10 years among patients diagnosed with CLL in 2003–2015 (2101 PYs). Furthermore, the elevated risk of SPMs was seen among patients >80-years-old, which was not apparent in the previous study periods (SIR 1.12, 95% CI: 0.93–1.34 in 1973–1982 vs. SIR 1.26, 95% CI: 1.13–1.4 in 2003–2015).

### Effect of treatment

Of the 38,754 CLL patients studied, 9988 (1640 SPMs) were listed as having received chemotherapy in the SEER database while the remaining 28,766 (4847 SPMs) were classified as not treated/unknown. Although the risk of developing SPMs as compared to the U.S. population was elevated among both these groups, it was higher among patients who received systemic treatment for CLL (SIR 1.38 95% CI: 1.31–1.44) vs. the not treated/unknown group (SIR 1.16, 95% CI: 1.13–1.19, *p* < 0.001). When stratified by study periods, the risk for SPMs increased among patients who received treatment (SIR 1.29, 95% CI: 1.17–1.43 in 1973–1982 vs. SIR 1.80, 95% CI: 1.61–2.01 during 2003–2015), as well as among those who were not treated (SIR 1.14, 95% CI: 1.05–1.22 in 1973–1982 vs. SIR 1.29, 95% CI: 1.23–1.35 during 2003–2015). In the more recent study period, there was no difference in the risk for overall solid tumors among the patients who received treatment (SIR 1.18, 95% CI: 1.12–1.28) as compared to those not treated (SIR 1.37, 95% CI: 1.19–1.56). Of note, the risk for prostate cancer was higher among the patients who did not receive treatment for CLL (SIR 1.15, 95% CI: 1.01–1.31 vs. SIR 0.91, 95% CI: 0.63–1.28). The risk for all hematological malignancies (SIR: 2.17, 95% CI: 1.92–2.46 vs. SIR: 5.36, 95% CI: 4.32–6.58), NHL (SIR: 2.98, 95% CI: 2.54–3.47 vs. SIR: 7.68, 95% CI: 5.89–9.85) and AML (SIR: 2.18, 95% CI: 1.45–3.82 vs. SIR: 9.81, 95% CI: 5.61–15.93) was significantly higher among the patients who received treatment as compared to those who did not. We also performed a separate analysis looking at patients who received chemotherapy, radiation therapy, both treatment modalities, or no therapy and looked at overall and site wise SIRs over time. Results were similar to the analysis for chemotherapy vs. untreated/unknown group (Supplementary Table 3).

**Fig. 4:**
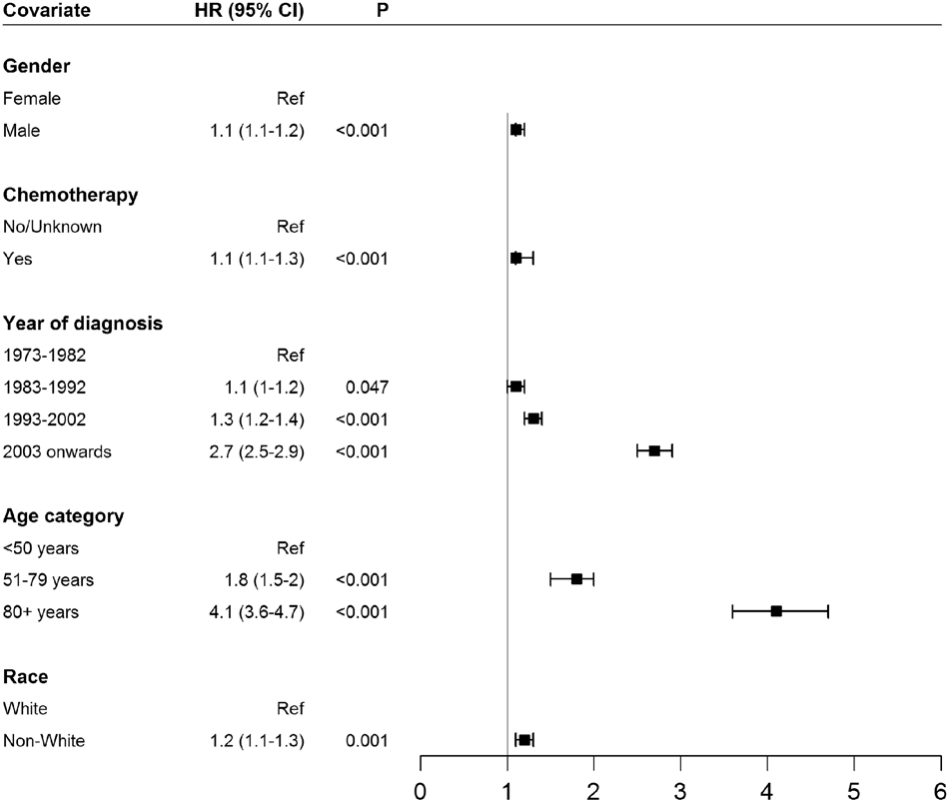
Hazard of developing second primary malignancies (SPMs) in patients with a diagnosis of chronic lymphocytic leukemia (CLL) in a multivariate model utilizing patient gender, age category at diagnosis, race, year of diagnosis, and whether or not the patients received chemotherapy

We performed a multivariate analysis for looking at the risk of developing SPMs in CLL patients using gender, age category, race, year of diagnosis, and chemotherapy treatment as the variables. The hazard of developing SPMs was higher in males (HR 1.118, *p* < 0.001) and for those who received chemotherapy (HR 1.234, *p* < 0.001). There was a significant and progressively increasing hazard of developing SPMs noted with the year of diagnosis range for CLL from before 1983, 1983–1992, 1993–2002 to 2003–2015. Similarly, a progressively increasing risk of SPMs was noted with advancing patient age at the time of CLL diagnosis and for non-White patients as compared to Whites (HR 1.172, *p* = 0.001) (Fig. 4).

**Fig. 5:**
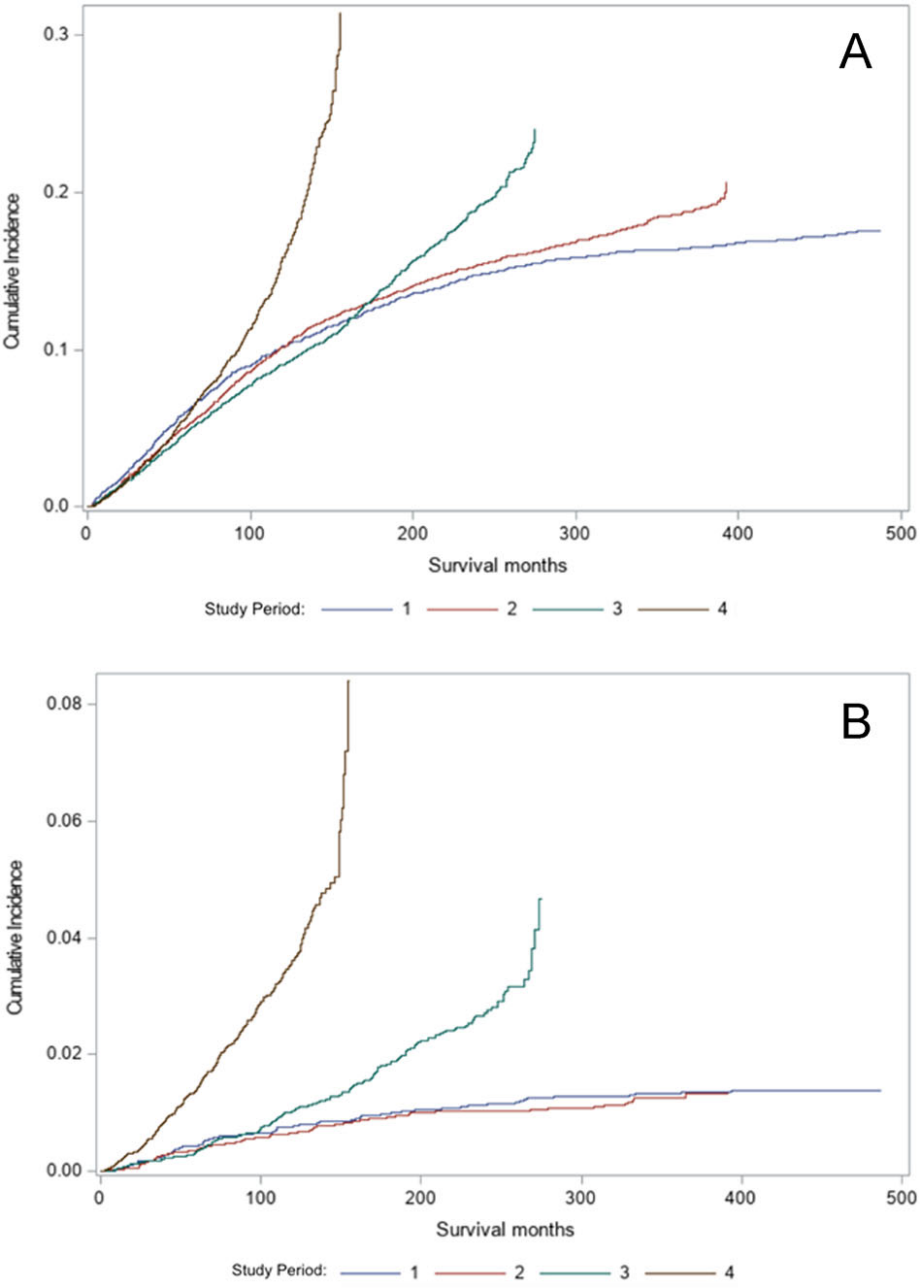
Cumulative incidence of second primary malignancies (SPMs) over time. Periods for **a** solid tumors and **b** all hematologic malignancies. Study periods include: (1) 1973–1982, (2) 1983–1992, (3) 1993–2002, and (4) 2003–2015

We performed a survival analysis of CLL patients with SPMs, but this analysis was somewhat nuanced. Patients who lived the longest had the highest cumulative incidence of SPMs, and thus, in the survival analysis it appeared that survival is superior among patients who develop SPMs, which is erroneous. Instead, due to the presence of a competing event (death), we compared the cumulative incidence of SPMs in different study periods among patients with CLL, which represents the absolute risk of developing any SPM (Table 3). It is evident that the cumulative incidence of SPMs was higher in the most recent study period at 60 months and onwards, consistent with the natural history and average survival of patients with CLL. The cumulative incidence of solid tumors was almost similar at 60 months, but risk increased at 120 months and afterwards (Fig. 5a), while the risk of hematological malignancies was higher across all the study periods (Fig. 5b).

**Table 3:** Cumulative incidence of second primary malignancies (SPMs) in patients with chronic lymphocytic leukemia (cll) stratified by study periods

	1973–1982	1983–1992	1993–2002	2003–2015
All sites				
12-months follow-up	1.2 (0.9–1.5)	0.8 (0.6–1.0)	0.8 (0.6–1.0)	0.9 (0.8–1.0)
60-months follow-up	6.6 (5.8–7.2)	5.5 (4.9–6.0)	5.1 (4.6–5.5)	7.1 (6.6–7.6)
120-months follow-up	11.1 (10.3–11.9)	10.8 (10.2–11.5)	10.1 (9.5–10.8)	19.5 (18.5–20.5)
All solid tumors				
12-months follow-up	1.2 (0.9–1.5)	0.8 (0.6–1)	0.8 (0.6–1)	0.7 (0.6–0.8)
60-months follow-up	6.1 (5.4–6.7)	5.1 (4.6–5.6)	4.7 (4.3–5.2)	5.7 (5.3–6.1)
120-months follow-up	10.3 (9.5–11.1)	10.1 (9.5–10.8)	9.1 (8.6–9.8)	15.9 (15–16.7)
All lymphatic and hematopoietic diseases				
12-months follow-up	0.05 (0.02–0.2)	0.04 (0.01–0.1)	0.05 (0.02–0.1)	0.2 (0.1–0.3)
60-months follow-up	0.5 (0.3–0.7)	0.4 (0.3–0.5)	0.4 (0.2–0.5)	1.4 (1.2–1.7)
120-months follow-up	0.8 (0.6–1.1)	0.7 (0.5–0.9)	1.0 (0.8–1.3)	3.6 (3.2–4.1)

## Discussion

Outcomes in CLL have been improving progressively, with better overall survival (OS) and thus, increasing prevalence. There have been several new therapeutic agents for CLL approved by the Food and Drug Administration (FDA) in recent years^14^. This has even resulted in a paradigm shift in the management of CLL, with a focus on newer targeted therapy, rather than the long-standing standard of chemoimmunotherapy^15^. It has been reported that diagnosis of a primary malignancy is associated with an increased risk of SPMs as compared to the general population without a cancer diagnosis^16^. In CLL, a malignancy typically associated with long survivorship, the risk of SPMs can be significant but temporal trends and association of SPMs with various patient and treatment characteristics have not been previously reported in a comprehensive analysis. We performed such an analysis from the large, population-based SEER database spanning over four decades, hopefully leading to optimal patient care.

We noted a significantly high rate of SPMs, both solid-organ and hematologic malignancies in patients with CLL as compared to age-matched U.S. population. This is similar to previously reported studies from France and Australia, but our analysis showed a differing rate of SPMs than has been previously reported, possibly due to a longer follow-up as well as heterogeneity in patient demographics and in patterns of environmental exposures among other factors^7,17,18^. We noticed a decreased risk for certain cancer types among CLL patients, which has not been previously reported by other studies. This was primarily for hepatobiliary, breast, and uterine cancers and could be due to specific genetic and environmental factors that play a role in these malignancies, hypothetically independent of CLL occurrence. We noted a higher risk of prostate cancer among CLL patients who did not receive treatment as compared to those who did. It is possible that patients not on active cancer treatment follow recommended cancer screening while patients who are on treatment for the CLL may not be following up on the recommended cancer screenings, including prostate cancer. This hypothesis is different from the observation that in case of some other primary malignancies, patients have been reported to have a higher incidence of SPMs, presumably due to being integrated in the healthcare system and undergoing increased screening^19^.

While standard ICD-O-3 diagnosis codes were used to identify the various SPMs, we considered that NHL diagnosis, particularly SLL may be overlapping with CLL. We performed additional analyses after excluding NHL as the SPM and still noted an increased risk for hematologic SPMs among CLL patients. It is notable that in other analyses as well, NHL is the most common SPM among CLL patients^20^. Furthermore, in a single-center experience, it was noted that CLL patients treated with the FCR regimen had a higher incidence of AML and NHL, possibly as a drug effect^11^. It has been standard practice in previous studies to not include SPMs within first 2 months after diagnosis of the primary malignancy to eliminate coexisting malignancies that may be diagnosed separately^13,21^. We noted the highest incidence of a SPM diagnosis to be between 2 and 6 months after CLL diagnosis. While this raises the possibility of ascertainment bias and the SPMs being diagnosed secondary to heightened medical attention due to CLL, this phenomenon cannot be assessed from population-based studies and could potentially be explored in large, institutional data. Nevertheless, increased SIR of SPMs was seen among CLL patients throughout the follow-up period. There was an increased incidence of SPMs for CLL patients in the 50–79 years age-group as compared to younger and older patients although when tested in a multivariate model, the risk of SPMs increased progressively with age. A previous single-center study has also mentioned age as an independent risk factor for developing SPMs^20^. Improving longevity of the US population as a whole and longer survival of CLL patients due to better tolerated therapeutic options even for the elderly may explain this. There was a variable risk of SPMs noted for patients of different racial-ethnic backgrounds, with a higher rate noted in the African–American minority. We have previously reported such disparate trends in multiple myeloma, another lymphoid malignancy as well^22^.

We noted an increasing trend of SPMs in more recent years. Such temporal trends have been reported in other studies focusing on various index malignancy diagnoses^23,24^. While causes for this phenomenon appear multifactorial, enhanced screening practices, better diagnostic tools, and availability of better tolerated therapeutic options that may be employed sooner in the disease course could explain at least some of it. In our analysis, the primary contributor for this trend was an increase in hematologic SPMs over time rather than solid-organ cancers, the risk for which remained relatively uniformly increased among CLL patients. Among the hematologic SPMs, NHL and AML were noted to increase more in recent years. This trend has been previously reported in a single-center experience with patients treated uniformly with the FCR regimen, involving agents that can induce DNA damage in hematopoietic stem cells over time^11^. The advent of more targeted therapies, that have been approved by the FDA recently, would not have been captured in our analysis. We do hypothesize that in future analyses, in case a decrease in initial FCR utilization is noted, there may be a decrease in SPMs as well among CLL patients. Curiously, we have previously reported an opposite trend of decreasing SPM risk over time in patients with primary HL^12^. In that case, the specific evolution in HL management with a decrease in the use of extensive radiation therapy and a resultant decrease in breast SPMs were noted as the biggest contributors to decreasing SPMs. We noted the highest SIRs for patients age 15–49 years although the highest absolute excess of SPMs was noted for the 50–79 age group. Previous studies have also reported that SPM SIRs are typically highest for individuals diagnosed at younger ages and decline thereafter^25^. Mechanisms of carcinogenesis may be variable for the different age groups, including the roles of contributing etiologic factors and interactions between influences (including gene-environment and gene-gene interactions). Cancer treatment for younger individuals is typically more aggressive and involves chemotherapy and/or radiation therapy, which may have a higher carcinogenic potential. Indeed, in recent years there have been more immunotherapy-based treatment approaches developed for CLL patients not considered candidates for traditional cytotoxic chemoimmunotherapy^26^. It is possible that since several patients with CLL may not receive treatment, despite the CLL diagnosis as against many other malignancies where treatment is initiated after the primary cancer diagnosis, screening practices for SPMs may be different for CLL patients receiving treatment vs. those who are only on surveillance.

Traditional anti-cancer therapeutics, especially cytotoxic chemotherapy have been associated with a risk of secondary cancers, including in the case of CLL^6–9,11,18,27–33^. This has been noted in data from clinical trials, single-institution experience, and limited population-based analyses. At the same time, some older analyses from population-based studies suggested that there may not be any association of SPMs in CLL and prior therapy^34^. In our analysis, while all CLL patients had a higher risk of SPMs than the general U.S. population, the risk was noted to be higher in patients who had been treated with prior systemic therapy. While the granular details about type and duration of treatment are not available from the SEER database, a longer follow-up in our analysis as compared to previous ones may have been the reason why a clear temporal trend across all time periods was noted. Potentially, claims-based data may be able to provide detailed treatment-related information to further address the link between specific treatment options and subsequent SPMs.

Identification of SPMs and their management is becoming an important part of survivorship in cancer patients, especially in diseases where therapeutic innovations have improved survival from the primary malignancy. This is true for CLL as well, where the availability of recent, targeted and well-tolerated treatments is making a difference in patient outcomes, even including those with very high-risk disease^35^. SPMs may reflect the effect of shared etiologic factors, environmental exposures, host characteristics, as well as an attribution to prior cancer treatment. We demonstrate trends in the incidence of SPMs among patients with CLL with a longer follow-up and a larger patient sample, thus building upon previously known data. This could inform further studies about the etiology of SPMs and help shape improved survivorship for patients with CLL.

## Supplementary information

Supplementary Tables

Reproducibility Checklist
