# Supplementary material for: Trends in the risk of second primary malignancies among survivors of chronic lymphocytic leukemia
Source: Blood Cancer J. 2019 Sep 30;9(10):75. doi: 10.1038/s41408-019-0237-1 (PMC6768881; doi:10.1038/s41408-019-0237-1)
Supplement: Supplementary file 1 — Supplementary Tables [file 41408_2019_237_MOESM1_ESM.docx]

**Supplementary Table 1.** Standardized Incidence Ratios (SIRs) of Second Primary Malignancies (SPMs) by Latency Period After the Diagnosis of Chronic Lymphocytic Leukemia (CLL) Over Time, Excluding SPMs that Occur Within First Year After the Diagnosis of CLL.

|  | 1973-2015 | 1973-2015 | 1973-2015 | 1973-1982 | 1973-1982 | 1973-1982 | 1983-1992 | 1983-1992 | 1983-1992 | 1993-2002 | 1993-2002 | 1993-2002 | 2003-2015 | 2003-2015 | 2003-2015 |
| --- | --- | --- | --- | --- | --- | --- | --- | --- | --- | --- | --- | --- | --- | --- | --- |
|  | **Observed** | **O/E** | **Excess Risk** | **Observed** | **O/E** | **Excess Risk** | **Observed** | **O/E** | **Excess Risk** | **Observed** | **O/E** | **Excess Risk** | **Observed** | **O/E** | **Excess Risk** |
| All Sites | 5,596 | 1.19# | 38.98 | 890 | 1.19# | 38.14 | 1,462 | 1.10# | 21.73 | 1,718 | 1.18# | 36.73 | 1,526 | 1.32# | 58.96 |
| All Sites excluding Non-Melanoma Skin | 5,519 | 1.18# | 36.57 | 881 | 1.18# | 36.24 | 1,442 | 1.09# | 19.24 | 1,694 | 1.17# | 34.4 | 1,502 | 1.30# | 56.24 |
| All Solid Tumors | 4,682 | 1.13# | 23.53 | 784 | 1.19# | 32.54 | 1,287 | 1.09# | 17.75 | 1,433 | 1.12# | 21.27 | 1,178 | 1.16# | 26.32 |
| Oral Cavity and Pharynx | 125 | 1.17 | 0.78 | 26 | 1.38 | 1.9 | 28 | 0.96 | -0.18 | 39 | 1.25 | 1.07 | 32 | 1.16 | 0.7 |
| Lip | 26 | 1.86# | 0.52 | 11 | 3.21# | 2.01 | 7 | 1.55 | 0.41 | 5 | 1.39 | 0.19 | 3 | 1.24 | 0.09 |
| Tongue | 19 | 0.72 | -0.31 | 2 | 0.56 | -0.41 | 3 | 0.49 | -0.52 | 6 | 0.73 | -0.3 | 8 | 0.96 | -0.05 |
| Salivary Gland | 38 | 3.14# | 1.11 | 5 | 2.93 | 0.87 | 8 | 2.49# | 0.79 | 16 | 4.14# | 1.69 | 9 | 2.70# | 0.91 |
| Floor of Mouth, and Gum and Other Mouth | 19 | 0.74 | -0.28 | 4 | 0.79 | -0.28 | 2 | 0.27# | -0.89 | 10 | 1.37 | 0.38 | 3 | 0.52 | -0.44 |
| Floor of Mouth | 6 | 0.8 | -0.06 | 1 | 0.56 | -0.21 | 1 | 0.42 | -0.22 | 3 | 1.53 | 0.14 | 1 | 0.74 | -0.06 |
| Gum and Other Mouth | 13 | 0.72 | -0.22 | 3 | 0.92 | -0.07 | 1 | 0.2 | -0.67 | 7 | 1.32 | 0.23 | 2 | 0.45 | -0.39 |
| Tonsil | 5 | 0.45 | -0.26 | 1 | 0.65 | -0.15 | 1 | 0.4 | -0.25 | 0 | 0 | -0.47 | 3 | 0.8 | -0.12 |
| Pharynx | 11 | 0.74 | -0.17 | 1 | 0.35 | -0.5 | 4 | 0.93 | -0.05 | 2 | 0.47 | -0.31 | 4 | 1.14 | 0.08 |
| Nasopharynx | 4 | 1.27 | 0.04 | 1 | 1.79 | 0.12 | 1 | 1.13 | 0.02 | 1 | 1.05 | 0.01 | 1 | 1.31 | 0.04 |
| Oropharynx | 2 | 0.6 | -0.06 | 0 | 0 | -0.13 | 0 | 0 | -0.13 | 0 | 0 | -0.14 | 2 | 1.92 | 0.15 |
| Hypopharynx | 5 | 0.59 | -0.15 | 0 | 0 | -0.48 | 3 | 1.15 | 0.06 | 1 | 0.44 | -0.18 | 1 | 0.59 | -0.11 |
| Other Oral Cavity and Pharynx | 7 | 2.34 | 0.17 | 2 | 3.09 | 0.36 | 3 | 3.04 | 0.33 | 0 | 0 | -0.11 | 2 | 3.34 | 0.23 |
| Digestive System | 1,020 | 1.04 | 1.55 | 197 | 1.08 | 4.06 | 301 | 1.05 | 2.55 | 318 | 1.08 | 3.39 | 204 | 0.91 | -3.08 |
| Esophagus | 48 | 0.87 | -0.31 | 9 | 1.16 | 0.32 | 13 | 0.88 | -0.29 | 14 | 0.79 | -0.53 | 12 | 0.81 | -0.46 |
| Stomach | 86 | 0.97 | -0.13 | 21 | 1.15 | 0.71 | 28 | 1.07 | 0.28 | 17 | 0.67 | -1.17 | 20 | 1.04 | 0.14 |
| Small Intestine | 18 | 0.94 | -0.05 | 4 | 1.79 | 0.47 | 4 | 0.87 | -0.1 | 5 | 0.8 | -0.17 | 5 | 0.83 | -0.16 |
| Colon, Rectum and Anus | 649 | 1.13# | 3.14 | 134 | 1.13 | 4.19 | 198 | 1.11 | 3.24 | 209 | 1.25# | 5.81 | 108 | 0.96 | -0.67 |
| Colon and Rectum | 632 | 1.12# | 2.9 | 133 | 1.14 | 4.32 | 196 | 1.12 | 3.36 | 203 | 1.24# | 5.48 | 100 | 0.92 | -1.39 |
| Colon excluding Rectum | 480 | 1.13# | 2.44 | 103 | 1.2 | 4.47 | 144 | 1.1 | 2.05 | 153 | 1.24# | 4.09 | 80 | 0.98 | -0.3 |
| Cecum | 149 | 1.46# | 2.01 | 33 | 1.62# | 3.34 | 45 | 1.42# | 2.17 | 54 | 1.79# | 3.32 | 17 | 0.86 | -0.46 |
| Appendix | 3 | 0.71 | -0.05 | 1 | 2.4 | 0.15 | 0 | 0 | -0.14 | 1 | 0.73 | -0.05 | 1 | 0.64 | -0.09 |
| Ascending Colon | 94 | 1.26# | 0.84 | 17 | 1.37 | 1.22 | 21 | 0.99 | -0.05 | 32 | 1.34 | 1.14 | 24 | 1.41 | 1.12 |
| Hepatic Flexure | 32 | 1.39 | 0.38 | 3 | 0.71 | -0.32 | 8 | 1.08 | 0.1 | 16 | 2.25# | 1.24 | 5 | 1.14 | 0.1 |
| Transverse Colon | 51 | 1.24 | 0.42 | 12 | 1.47 | 1.02 | 20 | 1.61 | 1.25 | 11 | 0.91 | -0.16 | 8 | 0.94 | -0.09 |
| Splenic Flexure | 18 | 1.23 | 0.15 | 4 | 1.22 | 0.19 | 8 | 1.63 | 0.51 | 3 | 0.74 | -0.15 | 3 | 1.25 | 0.1 |
| Descending Colon | 21 | 0.87 | -0.13 | 3 | 0.53 | -0.71 | 11 | 1.44 | 0.55 | 6 | 0.92 | -0.07 | 1 | 0.23 | -0.52 |
| Sigmoid Colon | 88 | 0.78# | -1.06 | 27 | 1.01 | 0.1 | 20 | 0.54# | -2.83 | 25 | 0.82 | -0.78 | 16 | 0.88 | -0.35 |
| Large Intestine, NOS | 24 | 0.9 | -0.11 | 3 | 0.6 | -0.53 | 11 | 1.38 | 0.5 | 5 | 0.63 | -0.41 | 5 | 0.88 | -0.11 |
| Rectum and Rectosigmoid Junction | 152 | 1.08 | 0.46 | 30 | 0.98 | -0.16 | 52 | 1.18 | 1.31 | 50 | 1.25 | 1.39 | 20 | 0.75 | -1.08 |
| Rectosigmoid Junction | 41 | 0.96 | -0.07 | 7 | 0.67 | -0.92 | 10 | 0.7 | -0.71 | 19 | 1.71# | 1.1 | 5 | 0.76 | -0.25 |
| Rectum | 111 | 1.12 | 0.52 | 23 | 1.14 | 0.77 | 42 | 1.41# | 2.02 | 31 | 1.07 | 0.3 | 15 | 0.74 | -0.83 |
| Anus, Anal Canal and Anorectum | 17 | 1.49 | 0.24 | 1 | 0.67 | -0.13 | 2 | 0.74 | -0.12 | 6 | 1.64 | 0.33 | 8 | 2.27 | 0.72 |
| Rectum, Rectosig Junct, Anus, Anal Canal and Anorectum | 169 | 1.11 | 0.7 | 31 | 0.97 | -0.28 | 54 | 1.15 | 1.19 | 56 | 1.28 | 1.72 | 28 | 0.93 | -0.36 |
| Liver, Gallbladder, Intrahep Bile Duct and Other Biliary | 67 | 0.74# | -1 | 6 | 0.52 | -1.44 | 18 | 0.84 | -0.58 | 21 | 0.73 | -1.09 | 22 | 0.77 | -1.03 |
| Liver | 32 | 0.66# | -0.7 | 3 | 0.62 | -0.48 | 5 | 0.48 | -0.89 | 10 | 0.63 | -0.82 | 14 | 0.82 | -0.5 |
| Gallbladder | 16 | 1.21 | 0.12 | 1 | 0.36 | -0.47 | 6 | 1.62 | 0.38 | 7 | 1.86 | 0.45 | 2 | 0.67 | -0.16 |
| Intrahep and Extrahep Bile Ducts, and Other Biliary | 19 | 0.66 | -0.42 | 2 | 0.52 | -0.49 | 7 | 0.95 | -0.06 | 4 | 0.44 | -0.72 | 6 | 0.72 | -0.38 |
| Intrahepatic Bile Duct | 6 | 0.68 | -0.12 | 0 | 0 | -0.24 | 1 | 0.43 | -0.22 | 2 | 0.71 | -0.11 | 3 | 1.07 | 0.03 |
| Other Biliary | 13 | 0.65 | -0.3 | 2 | 0.68 | -0.25 | 6 | 1.19 | 0.15 | 2 | 0.32 | -0.6 | 3 | 0.54 | -0.41 |
| Pancreas | 125 | 0.89 | -0.68 | 19 | 0.86 | -0.81 | 35 | 0.95 | -0.29 | 38 | 0.87 | -0.77 | 33 | 0.86 | -0.86 |
| Retroperitoneum | 4 | 1.22 | 0.03 | 0 | 0 | -0.15 | 2 | 2.29 | 0.18 | 2 | 2.01 | 0.14 | 0 | 0 | -0.13 |
| Peritoneum, Omentum and Mesentery | 6 | 1.55 | 0.09 | 0 | 0 | -0.07 | 0 | 0 | -0.14 | 5 | 3.29# | 0.49 | 1 | 0.83 | -0.03 |
| Other Digestive Organs | 17 | 2.60# | 0.45 | 4 | 4.89# | 0.84 | 3 | 2 | 0.25 | 7 | 3.31# | 0.68 | 3 | 1.43 | 0.15 |
| Respiratory System | 1,131 | 1.47# | 15.45 | 212 | 1.69# | 22.87 | 325 | 1.47# | 16.99 | 330 | 1.38# | 12.73 | 264 | 1.42# | 12.59 |
| Nose, Nasal Cavity and Middle Ear | 13 | 2.02# | 0.28 | 2 | 1.85 | 0.24 | 3 | 1.69 | 0.2 | 2 | 1.04 | 0.01 | 6 | 3.64# | 0.7 |
| Larynx | 58 | 1.41# | 0.73 | 12 | 1.48 | 1.04 | 24 | 1.93# | 1.9 | 12 | 1.04 | 0.06 | 10 | 1.11 | 0.16 |
| Pleura | 0 | 0 | -0.02 | 0 | 0 | -0.02 | 0 | 0 | -0.02 | 0 | 0 | -0.02 | 0 | 0 | -0.02 |
| Lung, Bronchus, Trachea, Mediastinum and Other Resp Org | 1,060 | 1.46# | 14.46 | 198 | 1.70# | 21.61 | 298 | 1.44# | 14.91 | 316 | 1.40# | 12.68 | 248 | 1.42# | 11.74 |
| Lung and Bronchus | 1,058 | 1.46# | 14.43 | 197 | 1.70# | 21.42 | 298 | 1.44# | 14.96 | 315 | 1.40# | 12.59 | 248 | 1.42# | 11.78 |
| Trachea | 1 | 1.48 | 0.01 | 0 | 0 | -0.04 | 0 | 0 | -0.03 | 1 | 5.43 | 0.11 | 0 | 0 | -0.02 |
| Mediastinum and Other Respiratory Organs | 1 | 1.8 | 0.02 | 1 | 8.27 | 0.23 | 0 | 0 | -0.03 | 0 | 0 | -0.02 | 0 | 0 | -0.02 |
| Bones and Joints | 5 | 1.3 | 0.05 | 1 | 1.64 | 0.1 | 2 | 2 | 0.16 | 1 | 0.84 | -0.03 | 1 | 0.95 | -0.01 |
| Soft Tissue including Heart | 39 | 1.73# | 0.71 | 6 | 2.12 | 0.84 | 7 | 1.25 | 0.23 | 11 | 1.49 | 0.5 | 15 | 2.24# | 1.34 |
| Skin excluding Basal and Squamous | 432 | 2.25# | 10.31 | 55 | 3.37# | 10.25 | 89 | 2.12# | 7.72 | 148 | 2.23# | 11.37 | 140 | 2.07# | 11.65 |
| Melanoma of the Skin | 355 | 2.07# | 7.9 | 46 | 3.17# | 8.34 | 69 | 1.86# | 5.23 | 124 | 2.10# | 9.04 | 116 | 1.92# | 8.92 |
| Other Non-Epithelial Skin | 77 | 3.67# | 2.41 | 9 | 4.95# | 1.9 | 20 | 4.13# | 2.49 | 24 | 3.30# | 2.33 | 24 | 3.39# | 2.72 |
| Breast | 376 | 0.90# | -1.82 | 59 | 0.84 | -2.88 | 107 | 0.93 | -1.42 | 115 | 0.89 | -1.92 | 95 | 0.91 | -1.44 |
| Female Breast | 364 | 0.88# | -2.05 | 59 | 0.86 | -2.65 | 104 | 0.91 | -1.63 | 107 | 0.84 | -2.74 | 94 | 0.92 | -1.29 |
| Male Breast | 12 | 1.8 | 0.23 | 0 | 0 | -0.23 | 3 | 1.74 | 0.21 | 8 | 3.73# | 0.82 | 1 | 0.52 | -0.15 |
| Female Genital System | 141 | 0.85 | -1.07 | 24 | 0.76 | -1.98 | 34 | 0.75 | -1.9 | 49 | 1 | 0.01 | 34 | 0.85 | -0.95 |
| Cervix Uteri | 10 | 0.83 | -0.09 | 2 | 0.64 | -0.3 | 2 | 0.56 | -0.26 | 6 | 1.94 | 0.41 | 0 | 0 | -0.37 |
| Corpus and Uterus, NOS | 68 | 0.77# | -0.86 | 10 | 0.61 | -1.68 | 18 | 0.76 | -0.93 | 20 | 0.77 | -0.83 | 20 | 0.91 | -0.33 |
| Corpus Uteri | 68 | 0.79 | -0.76 | 10 | 0.63 | -1.58 | 18 | 0.78 | -0.83 | 20 | 0.79 | -0.73 | 20 | 0.93 | -0.23 |
| Uterus, NOS | 0 | 0 | -0.1 | 0 | 0 | -0.11 | 0 | 0 | -0.09 | 0 | 0 | -0.1 | 0 | 0 | -0.11 |
| Ovary | 46 | 0.96 | -0.09 | 10 | 1.1 | 0.24 | 8 | 0.58 | -0.95 | 16 | 1.1 | 0.21 | 12 | 1.12 | 0.2 |
| Vagina | 1 | 0.32 | -0.09 | 0 | 0 | -0.16 | 0 | 0 | -0.14 | 1 | 1.07 | 0.01 | 0 | 0 | -0.12 |
| Vulva | 14 | 1.25 | 0.12 | 2 | 1.07 | 0.04 | 6 | 1.99 | 0.49 | 4 | 1.15 | 0.07 | 2 | 0.69 | -0.14 |
| Other Female Genital Organs | 2 | 0.6 | -0.06 | 0 | 0 | -0.11 | 0 | 0 | -0.12 | 2 | 2.02 | 0.14 | 0 | 0 | -0.19 |
| Male Genital System | 880 | 0.94 | -2.41 | 125 | 0.9 | -3.55 | 271 | 0.92 | -3.61 | 256 | 0.87# | -5.32 | 228 | 1.08 | 2.82 |
| Prostate | 864 | 0.93# | -2.7 | 122 | 0.89 | -3.98 | 268 | 0.92 | -3.72 | 251 | 0.86# | -5.62 | 223 | 1.07 | 2.43 |
| Testis | 3 | 1.21 | 0.02 | 1 | 2.85 | 0.17 | 0 | 0 | -0.09 | 0 | 0 | -0.11 | 2 | 2.58 | 0.2 |
| Penis | 9 | 1.82 | 0.17 | 2 | 2.52 | 0.32 | 2 | 1.51 | 0.11 | 3 | 1.99 | 0.21 | 2 | 1.5 | 0.11 |
| Other Male Genital Organs | 4 | 2.37 | 0.1 | 0 | 0 | -0.07 | 1 | 2.19 | 0.09 | 2 | 3.78 | 0.21 | 1 | 2.22 | 0.09 |
| Urinary System | 475 | 1.06 | 1.21 | 69 | 1.1 | 1.6 | 118 | 1 | -0.01 | 137 | 0.96 | -0.8 | 151 | 1.23# | 4.49 |
| Urinary Bladder | 316 | 1.03 | 0.39 | 51 | 1.13 | 1.53 | 81 | 0.97 | -0.4 | 87 | 0.89 | -1.43 | 97 | 1.2 | 2.58 |
| Kidney and Renal Pelvis | 141 | 1.11 | 0.62 | 13 | 0.84 | -0.67 | 32 | 1.03 | 0.16 | 47 | 1.13 | 0.78 | 49 | 1.27 | 1.68 |
| Renal Pelvis, Ureter and Other Urinary Organs | 29 | 1.11 | 0.12 | 6 | 1.4 | 0.45 | 6 | 0.86 | -0.16 | 9 | 1.12 | 0.14 | 8 | 1.15 | 0.17 |
| Kidney | 130 | 1.14 | 0.7 | 12 | 0.89 | -0.38 | 31 | 1.12 | 0.55 | 41 | 1.09 | 0.49 | 46 | 1.31 | 1.74 |
| Renal Pelvis | 11 | 0.86 | -0.08 | 1 | 0.48 | -0.29 | 1 | 0.29 | -0.4 | 6 | 1.53 | 0.29 | 3 | 0.89 | -0.06 |
| Ureter | 9 | 1.06 | 0.02 | 3 | 2.09 | 0.42 | 2 | 0.87 | -0.05 | 2 | 0.77 | -0.08 | 2 | 0.94 | -0.02 |
| Other Urinary Organs | 9 | 1.81 | 0.17 | 2 | 2.58 | 0.32 | 3 | 2.35 | 0.28 | 1 | 0.68 | -0.07 | 3 | 2.08 | 0.25 |
| Eye and Orbit | 9 | 1.25 | 0.08 | 3 | 2.73 | 0.5 | 0 | 0 | -0.31 | 4 | 1.78 | 0.25 | 2 | 1.02 | 0.01 |
| Eye and Orbit - Non-Melanoma | 1 | 0.71 | -0.02 | 0 | 0 | -0.06 | 0 | 0 | -0.06 | 0 | 0 | -0.06 | 1 | 2.63 | 0.1 |
| Eye and Orbit - Melanoma | 8 | 1.38 | 0.1 | 3 | 3.42 | 0.56 | 0 | 0 | -0.25 | 4 | 2.2 | 0.3 | 1 | 0.63 | -0.09 |
| Brain and Other Nervous System | 47 | 1.04 | 0.07 | 9 | 1.28 | 0.53 | 9 | 0.73 | -0.55 | 16 | 1.13 | 0.26 | 13 | 1.1 | 0.19 |
| Brain | 44 | 1.01 | 0.02 | 8 | 1.19 | 0.34 | 7 | 0.59 | -0.79 | 16 | 1.18 | 0.34 | 13 | 1.14 | 0.26 |
| Cranial Nerves Other Nervous System | 3 | 1.7 | 0.05 | 1 | 3.53 | 0.19 | 2 | 4.01 | 0.25 | 0 | 0 | -0.08 | 0 | 0 | -0.07 |
| Endocrine System | 49 | 1.35 | 0.55 | 2 | 0.59 | -0.37 | 9 | 1.28 | 0.33 | 22 | 1.82# | 1.38 | 16 | 1.16 | 0.36 |
| Thyroid | 45 | 1.37 | 0.52 | 2 | 0.68 | -0.25 | 7 | 1.13 | 0.14 | 20 | 1.82# | 1.26 | 16 | 1.25 | 0.52 |
| Thymus, Adrenal Gland and Other Endocrine | 4 | 1.18 | 0.03 | 0 | 0 | -0.12 | 2 | 2.38 | 0.19 | 2 | 1.79 | 0.12 | 0 | 0 | -0.16 |
| Thymus | 2 | 1.16 | 0.01 | 0 | 0 | -0.05 | 1 | 2.64 | 0.1 | 1 | 1.65 | 0.05 | 0 | 0 | -0.09 |
| Adrenal Gland | 0 | 0 | -0.05 | 0 | 0 | -0.06 | 0 | 0 | -0.06 | 0 | 0 | -0.05 | 0 | 0 | -0.05 |
| Other Endocrine | 2 | 4.85 | 0.07 | 0 | 0 | -0.02 | 1 | 9.48 | 0.15 | 1 | 8.1 | 0.12 | 0 | 0 | -0.02 |
| All Lymphatic and Hematopoietic Diseases | 677 | 1.63# | 11.25 | 64 | 1.09 | 1.47 | 99 | 0.91 | -1.67 | 220 | 1.65# | 12.07 | 294 | 2.57# | 28.88 |
| Lymphoma | 499 | 2.48# | 12.79 | 54 | 2.06# | 7.36 | 70 | 1.33# | 2.88 | 163 | 2.45# | 13.47 | 212 | 3.76# | 25.03 |
| Hodgkin Lymphoma | 85 | 8.93# | 3.25 | 12 | 7.42# | 2.75 | 18 | 7.17# | 2.54 | 37 | 12.70# | 4.75 | 18 | 7.27# | 2.5 |
| Hodgkin - Nodal | 79 | 8.63# | 3 | 11 | 7.03# | 2.5 | 17 | 7.03# | 2.4 | 34 | 12.20# | 4.35 | 17 | 7.14# | 2.35 |
| Hodgkin - Extranodal | 6 | 16.33# | 0.24 | 1 | 19.19 | 0.25 | 1 | 10.52 | 0.15 | 3 | 23.81# | 0.4 | 1 | 10.62 | 0.15 |
| Non-Hodgkin Lymphoma | 414 | 2.16# | 9.55 | 42 | 1.71# | 4.61 | 52 | 1.04 | 0.33 | 126 | 1.98# | 8.72 | 194 | 3.60# | 22.53 |
| NHL - Nodal | 273 | 2.09# | 6.14 | 29 | 1.60# | 2.9 | 30 | 0.87 | -0.76 | 85 | 2.00# | 5.92 | 129 | 3.68# | 15.11 |
| NHL - Extranodal | 141 | 2.29# | 3.41 | 13 | 1.99# | 1.71 | 22 | 1.44 | 1.1 | 41 | 1.96# | 2.8 | 65 | 3.44# | 7.42 |
| Myeloma | 51 | 0.74# | -0.78 | 6 | 0.6 | -1.06 | 14 | 0.78 | -0.66 | 14 | 0.65 | -1.06 | 17 | 0.87 | -0.4 |
| Leukemia | 127 | 0.88 | -0.77 | 4 | 0.18# | -4.83 | 15 | 0.39# | -3.88 | 43 | 0.95 | -0.35 | 65 | 1.69# | 4.25 |
| Lymphocytic Leukemia | 29 | 0.39# | -1.91 | 0 | 0.00# | -2.84 | 1 | 0.05# | -2.96 | 11 | 0.47# | -1.74 | 17 | 0.84 | -0.51 |
| Acute Lymphocytic Leukemia | 10 | 3.01# | 0.29 | 0 | 0 | -0.14 | 1 | 1.11 | 0.02 | 6 | 6.12# | 0.7 | 3 | 3.33 | 0.34 |
| Chronic Lymphocytic Leukemia | 8 | 0.12# | -2.48 | 0 | 0.00# | -2.49 | 0 | 0.00# | -2.77 | 3 | 0.14# | -2.53 | 5 | 0.27# | -2.13 |
| Other Lymphocytic Leukemia | 11 | 2.45# | 0.28 | 0 | 0 | -0.21 | 0 | 0 | -0.21 | 2 | 1.47 | 0.09 | 9 | 8.62# | 1.28 |
| Non-Lymphocytic Leukemia | 98 | 1.37# | 1.14 | 4 | 0.35# | -1.99 | 14 | 0.71 | -0.92 | 32 | 1.46 | 1.4 | 48 | 2.61# | 4.76 |
| Acute Non-Lymphocytic Leukemia (ANLL) | 67 | 1.47# | 0.92 | 1 | 0.14# | -1.57 | 7 | 0.57 | -0.86 | 22 | 1.56 | 1.1 | 37 | 3.00# | 3.97 |
| Myeloid and Monocytic Leukemia | 89 | 1.45# | 1.19 | 3 | 0.32# | -1.67 | 13 | 0.81 | -0.51 | 27 | 1.41 | 1.1 | 46 | 2.74# | 4.7 |
| Acute Myeloid Leukemia | 59 | 1.54# | 0.89 | 1 | 0.19 | -1.15 | 6 | 0.62 | -0.61 | 18 | 1.48 | 0.81 | 34 | 3.05# | 3.67 |
| Acute Monocytic Leukemia | 3 | 1.38 | 0.04 | 0 | 0 | -0.1 | 0 | 0 | -0.1 | 1 | 1.46 | 0.04 | 2 | 4.12 | 0.24 |
| Chronic Myeloid Leukemia | 22 | 1.2 | 0.16 | 2 | 0.69 | -0.24 | 4 | 0.79 | -0.17 | 7 | 1.24 | 0.19 | 9 | 1.92 | 0.69 |
| Other Myeloid/Monocytic Leukemia | 5 | 2.06 | 0.11 | 0 | 0 | -0.18 | 3 | 4.47 | 0.38 | 1 | 1.59 | 0.05 | 1 | 2.33 | 0.09 |
| Other Leukemia | 9 | 0.89 | -0.05 | 1 | 0.45 | -0.32 | 1 | 0.29 | -0.41 | 5 | 1.76 | 0.3 | 2 | 1.24 | 0.06 |
| Other Acute Leukemia | 5 | 1 | 0 | 0 | 0 | -0.32 | 1 | 0.54 | -0.14 | 3 | 2.42 | 0.25 | 1 | 1.46 | 0.05 |
| Aleukemic, Subleukemic and NOS | 4 | 0.78 | -0.05 | 1 | 1.03 | 0.01 | 0 | 0 | -0.27 | 2 | 1.25 | 0.06 | 1 | 1.07 | 0.01 |
| Mesothelioma | 18 | 1.17 | 0.11 | 2 | 0.94 | -0.03 | 4 | 0.91 | -0.06 | 7 | 1.41 | 0.29 | 5 | 1.28 | 0.17 |
| Kaposi Sarcoma | 12 | 3.36# | 0.36 | 3 | 4.21 | 0.61 | 3 | 2.4 | 0.29 | 4 | 4.31# | 0.43 | 2 | 2.93 | 0.21 |
| Miscellaneous | 160 | 1.35# | 1.8 | 33 | 1.34 | 2.23 | 56 | 1.52# | 3.16 | 41 | 1.23 | 1.06 | 30 | 1.28 | 1.05 |

Excess Risk is per 10,000, #=p<0.05

**Supplementary Table 2.** Risks of Secondary Malignancies Over the Study Period (1973-2015) in Chronic Lymphocytic Leukemia (CLL) Survivors within “Other” Races Including Asians, Native Americans and Pacific Islanders.

|  | Asians | | | Native Americans | | | Pacific Islanders | | | |
| --- | --- | --- | --- | --- | --- | --- | --- | --- | --- | --- |
|  | **Observed** | **O/E** | **Excess Risk** | **Observed** | **O/E** | **Excess Risk** | | **Observed** | **O/E** | **Excess Risk** |
| All Sites* | 81 | 1.52# | 73.07 | 17 | 2.45# | 170.86 | | 34 | 2.32# | 157.9 |
| All Solid Tumors | 63 | 1.32# | 40.74 | 17 | 2.73# | 182.79 | | 32 | 2.43# | 153.64 |
| All Lymphatic and Hematopoietic Diseases | 16 | 3.71# | 31.02 | 0 | 0 | -8.98 | | 2 | 1.77 | 7.12 |

*Includes miscellaneous sites as per the SEER database, not included in “solid tumors” and “lymphatic and hematopoietic diseases”.

Excess Risk is per 10,000, #=p<0.05

**Supplementary Table 3.** Risks of Secondary Malignancies Over the Study Period (1973-2015) in Chronic Lymphocytic Leukemia (CLL) Survivors who had Received Chemotherapy, Radiation Therapy, Both Modalities or with Untreated/Unknown Treatment Status.

|  | Chemotherapy | | Radiation Therapy | | Chemotherapy and Radiation Therapy | | Untreated/Unknown Treatment | |
| --- | --- | --- | --- | --- | --- | --- | --- | --- |
|  | **Observed** | **O/E** | **Observed** | **O/E** | **Observed** | **O/E** | **Observed** | **O/E** |
| All Sites* | 1,556 | 1.38# | 49 | 1.59# | 34 | 1.93# | 4,704 | 1.16# |
| All Solid Tumors | 1,264 | 1.26# | 38 | 1.39 | 25 | 1.59# | 3,999 | 1.12# |
| All Lymphatic and Hematopoietic Diseases | 226 | 2.42# | 7 | 2.78# | 9 | 6.32# | 506 | 1.39# |

*Includes miscellaneous sites as per the SEER database, not included in “solid tumors” and “lymphatic and hematopoietic diseases”.

Excess Risk is per 10,000, #=p<0.05
